# Stromal cell inhibition of anti-CD20 antibody mediated killing of B-cell malignancies

**DOI:** 10.3389/fcell.2023.1270398

**Published:** 2023-10-31

**Authors:** Ester Fagnano, Swati Pendharkar, Madyson Colton, Philip N. Jones, Marta Crespi Sallan, Tetyana Klymenko, Andrejs Braun, Christian Klein, Jamie Honeychurch, Eleanor J. Cheadle, Timothy M. Illidge

**Affiliations:** ^1^ Targeted Therapy Group, Division of Cancer Sciences, The University of Manchester, Manchester, United Kingdom; ^2^ Centre for Haemato-Oncology, John Vane Science Centre, Barts Cancer Institute, Queen Mary University of London, London, United Kingdom; ^3^ Department of Biosciences and Chemistry, Sheffield Hallam University, Sheffield, United Kingdom; ^4^ Roche Innovation Center Zurch, Roche Glycart AG, Schlieren, Switzerland

**Keywords:** CD20, antibody, lymphoma, tumor microenvironment, stroma

## Abstract

**Introduction:** The glycoengineered type II anti-CD20 monoclonal antibody obinutuzumab has been licensed for treatment in follicular non-Hodgkin lymphoma and B-CLL following clinical trials demonstrating superior outcomes to standard of care treatment. However, ultimately many patients still relapse, highlighting the need to understand the mechanisms behind treatment failure to improve patient care. Resistance to chemotherapy is often caused by the ability of malignant B-cells to migrate to the bone marrow and home into the stromal layer. Therefore, this study aimed to investigate whether stromal cells were also able to inhibit type II anti-CD20 antibody mechanisms of action, contributing to resistance to therapy.

**Methods:** A stromal-tumor co-culture was established *in vitro* between Raji or Daudi B-cell tumor cells and M210B4 stromal cells in 24 well plates.

**Results:** Contact with stromal cells was able to protect tumor cells from obinutuzumab mediated programmed cell death (PCD), antibody dependent cellular phagocytosis and antibody dependent cellular cytotoxicity. Furthermore, such protection required direct contact between stroma and tumor cells. Stromal cells appeared to interfere with obinutuzumab mediated B-cell homotypic adhesion through inhibiting and reversing actin remodelling, potentially as a result of stromal-tumor cell contact leading to downregulation of CD20 on the surface of tumor cells. Further evidence for the potential role of CD20 downregulation comes through the reduction in surface CD20 expression and inhibition of obinutuzumab mediated PCD when tumor cells are treated with Ibrutinib in the presence of stromal cells. The proteomic analysis of tumor cells after contact with stromal cells led to the identification of a number of altered pathways including those involved in cell adhesion and the actin cytoskeleton and remodeling.

**Discussion:** This work demonstrates that contact between tumor cells and stromal cells leads to inhibition of Obinutuzumab effector functions and has important implications for future therapies to improve outcomes to anti-CD20 antibodies. A deeper understanding of how anti-CD20 antibodies interact with stromal cells could prove a useful tool to define better strategies to target the micro-environment and ultimately improve patient outcomes in B-cell malignancies.

## 1 Introduction

B cell malignancies include those cancers that arise in B lymphocytes, such as B-cell non-Hodgkin’s lymphomas (B-NHL) and B-cell chronic lymphocytic leukaemias (B-CLL). B-NHL is the seventh most common cancer in the United States (as per 2018 projections (National Cancer Institute, 2020a; National Cancer Institute, 2020b)), and 74,000 new cases are reported every year (National Cancer Institute, 2020b). Since the introduction of the anti-CD20 monoclonal antibody (mAb) rituximab in combination with standard chemotherapeutic agents, mortality rates for B-NHL have significantly dropped (National Cancer Institute, 2012). Even better outcomes have been observed with the novel anti-CD20 mAb obinutuzumab: its use in a phase III clinical trial in patients with B-cell chronic lymphocytic leukemia (B-CLL) in combination with chlorambucil led to improved overall survival, progression-free survival and higher complete response rates than rituximab plus chlorambucil (Goede et al., 2014). In general, mAb targeting CD20 work through four main mechanisms of action: induction of programmed cell death (PCD), immune effector cell-mediated antibody-dependent cellular phagocytosis (ADCP) and antibody-dependent cellular cytotoxicity (ADCC), and complement-dependent cytotoxicity (CDC). PCD has been recently characterised, and involves the homotypic adhesion (HA) of B cells upon treatment, followed by reorganisation of the actin cytoskeleton and the movement of F-actin towards the cell-cell junction points (Ivanov et al., 2009; Alduaij et al., 2011). This process triggers the release of lysosomal content (lysosomal membrane permeabilisation, LMP), such as cathepsin B, which in turn leads to NAPDH oxidase-dependent generation of reactive oxygen species (ROS) and cell death (Ivanov et al., 2009; Honeychurch et al., 2012). Whilst type I mAbs such as rituximab strongly induce CDC but not PCD, type II mAbs such as obinutuzumab or tositumomab have greater ability at inducing PCD (Herter et al., 2013). Obinutuzumab is also specifically engineered in its Fc region to have an increased binding to Fc receptors on immune effector cells and thus is more effective at triggering ADCP and ADCC compared to rituximab and other mAbs (Mössner et al., 2010; Herter et al., 2014). However, despite the improvements in treatment outcomes obtained with the development of potent anti-CD20 mAbs, relapse is still common. One possible cause of relapse could be the protective tumour microenvironment (TME) in the bone marrow. In fact, leukemic cell (e.g., B-CLL, B-ALL) proliferation was found to be strictly dependent on the presence of TME components *in vitro* (Manabe et al., 1994; Lagneaux et al., 1998) and co-culture of B-CLL cells with stromal cells was also shown to provide resistance to standard chemotherapy (Panayiotidis et al., 1996; de la Fuente et al., 2002; Kurtova et al., 2009a). Similar observations were made in mantle cell lymphoma (MCL), where the presence of stromal components conferred protection from drug-induced apoptosis, possibly via p42/p44 MAPK pathway activation (Kurtova et al., 2009b). In diffuse large B-cell lymphoma (DLBCL), culturing human B cells in the presence of the stromal cell line HS-5 protected malignant cells from treatment with mitoxantrone via NFκB upregulation and subsequent inhibition of apoptosis. This effect, although lessened, was also observed in the absence of direct contact between tumour and stromal cells (Lwin et al., 2007). More recently, a protective effect of the TME on malignant B cells has been observed upon treatment with rituximab, and several therapeutic strategies, such as VLA-4 integrin blockade or inhibition of the CXCR-4/CXCL-12 signalling axis, have been developed to abrogate this protection (Buchner et al., 2010; Mraz et al., 2011; Hu et al., 2012; Beider et al., 2013). However, to date no studies have been performed in order to investigate whether the TME can influence the efficacy of type II mAb such as obinutuzumab-mediated killing of malignant B cells. Therefore, we sought to determine whether a stromal microenvironment protects against obinutuzumab-mediated B-cell killing. Our findings indicate that contact between stromal fibroblasts and B-NHL cell lines protects the latter from obinutuzumab-induced PCD, ADCC and ADCP. Interestingly, such a protective effect does not appear to be mediated by soluble factors, but rather by the direct contact between stromal and B-NHL tumour cells. A potential mechanism of protection from obinutuzumab-induced PCD appears to be related to the ability of stromal cells to downregulate CD20 and inhibit and reverse HA and actin remodelling that are caused by obinutuzumab treatment and are known to trigger the downstream signalling pathways leading to PCD (Honeychurch et al., 2012). Additionally, contact with stromal cells in the presence of the BTK inhibitor Ibrutinib further decreased obinutuzumab mediated PCD. A large-scale proteomic analysis highlighted several pathways which are differentially expressed in B-NHL cells upon culture on stromal cells and could potentially be targeted in order to abrogate resistance to treatment with obinutuzumab in patients with B cell malignancies. This work should aid the development of future therapeutic strategies which will enhance antibody-mediated tumour cell killing in protected niches throughout the body including the bone marrow and reduce the risk of relapse for patients with B cell malignancies.

## 2 Materials and Methods

### 2.1 Cell lines

B-cell lymphoma cell lines Raji and Daudi (Burkitt’s lymphoma) were purchased from American Type Culture Collection (ATCC). Murine bone marrow stromal cell line M2-10B4 was kindly provided by Claire Hart (University of Manchester, United Kingdom). Human bone marrow stromal cell line HS-5 and human embryonic kidney stromal cell line HEK293T were obtained from ATCC. Raji-GFP-actin cells (transfected to express a GFP-actin fusion protein) were generated by Dr Andrejs Ivanov (University of Manchester, United Kingdom) as described in Ivanov et al. (2009). Raji-YFP-CD20 cells (transfected to express a YFP-CD20 fusion protein) were kindly provided by Dr Andrejs Ivanov. All tumour cells and M2-10B4 stromal cells were grown under standard conditions (5% CO_2_, 37°C) in RPMI 1640 media (Gibco, Life Technology) supplemented with 10% heat-inactivated Foetal Bovine Serum (Invitrogen, Life Technologies, Thermo Fisher Scientific) and 2 mM L-glutamine (Invitrogen). HS-5 stromal cells were grown under standard conditions (5% CO_2_, 37°C) in DMEM media supplemented with 10% FBS and 2 mM L-glutamine. Cells were routinely screened to confirm negativity to *mycoplasma* infection.

### 2.2 Co-cultures

Murine M2-10B4 or human HS-5 bone marrow stromal cells were labelled with the fluorescence linker for general cell membrane labelling PKH67 (Sigma-Aldrich), following the manufacturer’s instructions, and plated in either 24- or 96-well plates (2.5 × 10^4^ cells/well or 5 × 10^3^ cells/well, respectively) until confluency (72 h approx.). Culture media were replaced with fresh media containing tumour cells at either 1.25 × 10^5^ cells/well or 2.5 × 10^4^ cells/well (24- and 96-well plates, respectively) and cells were co-cultured for 24 h. For stroma-conditioned media, M2-10B4 bone marrow stromal cells were plated in 24-well plates for 72 h and media were collected and centrifuged. The resulting supernatant was filtered through a 0.45 μm filter (Appleton Woods Ltd.) and used to culture tumour cells for 24 h. For tumour/stroma-conditioned media, M2-10B4 bone marrow stromal cells were plated in 24-well plates for 72 h. Tumour cells were added for additional 24 h, before being harvested and centrifuged. The resulting supernatant was filtered through a 0.45 μm filter and used to culture fresh tumour cells for 24 h. For non-contact transwell assays, 24-well polycarbonate transwell plates containing inserts of 0.4 μm pore size (Appleton Woods) were used. M2-10B4 stromal cells were plated into the bottom compartment (2.5 × 10^4^ cells/well) until confluency and tumour cells were added in the upper compartment (1 × 10^5^ cells/insert, in 100 μL media) for 24 h. For culture on fibronectin-coated wells, human fibronectin (Thermo Fisher Scientific) was added to 24-well plate wells at 5 μg/cm^2^ and incubated for 1 h at room temperature. The wells were then rinsed with distilled H_2_O and used to plate cells for 24 h.

### 2.3 Antibodies and reagents

Anti-CD20 monoclonal antibody obinutuzumab was provided by Dr Christian Klein (Roche Innovation Centre, Zurich, Switzerland). Anti-Her2 monoclonal antibody Herceptin was purchased from The Christie Hospital NHS Trust (Manchester, United Kingdom). Anti-human CD11b-APC antibody, anti-human CD20 APC, mouse IgG2b APC, anti-human CD56-APC antibody and anti-IFN-γ-PE antibody were obtained from eBioscience, Thermo Fisher Scientific. The Bruton’s tyrosine kinase inhibitor PCI-32765 (ibrutinib) was purchased from ApexBio Technology. Clones for each antibody can be found in Supplementary Material.

### 2.4 Generation of mCherry-M2-10B4 cell line

An mCherry lentiviral vector, containing an ampicillin-resistant gene under the Amp-R promoter and an mCherry-encoding gene under the SFFV promoter, was kindly provided by Dr Tiziana Monteverde (Cancer Research UK Manchester Institute, Manchester, United Kingdom). HEK293T cells (3 × 10^6^ cells/10 cm dish) were transfected with SFFV-mCherry plasmid (10 μg) plus the packaging plasmids pΔ19.8 (6.5 μg) and pMNG.2 (3.5 μg), provided by Dr Claire Dempsey, University of Manchester, United Kingdom, using 50 μL CaCl_2_. Lentiviral supernatant was collected 48 and 72 h later from transfected HEK293T cells and used to infect M2-10B4 cells by co-culture for 48 h, in the presence of 4 μg/mL polybrene (Sigma-Aldrich). Transduced cells were purified using FACS sorting.

### 2.5 Isolation of immune effector cells

Buffy coats of blood from healthy donors were purchased from Manchester Plymouth Grove Blood Donor Centre, Manchester, United Kingdom. Blood was obtained with ethical consent from the South Manchester Ethics committee in accordance with the declaration of Helsinki. Peripheral blood mononuclear cells (PBMCs) were extracted from buffy coats as described in Supplementary Material. Human monocytes were isolated from PBMCs by using a pan monocyte isolation kit (Miltenyi Biotec) following the manufacturer’s instructions. Human NK cells were isolated by using an NK cell isolation kit (Miltenyi Biotec) following the manufacturer’s instructions. Human macrophages were differentiated from monocytes by adding 50 μg/mL recombinant human M-CSF (macrophage colony-stimulating factor, Bio-Rad Laboratories Ltd.) on day 0 and day 4 to the culture media and growing them for 6 days.

### 2.6 Measurement of cell viability and NK cell activation

For programmed cell death, cells were harvested, washed in FACS buffer (PBS +1% FBS) and re-suspended in 0.5 μg/mL 7-Aminoactinomycin D (7-AAD, eBioscience) and 1 μg/mL AnnexinV-Cy5.5 (Becton-Dickinson). Samples were analysed by flow cytometry and percentages of viable tumour cells were calculated by measuring the percentage of cells negative to 7-AAD and AnnexinV, after excluding the PKH67+ population.

For antibody-dependent cellular phagocytosis (ADCP), tumour cells were labelled with the red fluorescence linker for general cell membrane labelling PKH26 (Sigma-Aldrich), following the manufacturer’s instructions. Human monocytes were isolated as described previously and added to the co-culture system at an effector to target ratio of 1 to 1 for 2 h. Cells were then harvested, washed in FACS buffer and labelled with an anti-CD11b-APC antibody. Samples were analysed by flow cytometry and cell viability was measured as percentage of cells positive to PKH26 and negative to CD11b, after excluding the PKH67+ population.

For antibody-dependent cellular cytotoxicity (ADCC), human NK cells were isolated as described previously, treated with an inhibitor of intracellular protein transport (Brefeldin A solution 1000X, eBioscience), and added to the co-culture system at an effector to target ratio of 1 to 1 for 4 h. Cells were then harvested, washed in FACS buffer and labelled with an anti-CD56-APC antibody. Cells were then fixed and permeabilised using a Foxp3/Transcription Factor Staining Buffer Set (eBioscience), following the manufacturer’s instructions, and intracellularly labelled with an anti-IFN-γ-PE antibody. Samples were analysed by flow cytometry and activation of NK cells was measured as percentage of cells positive to both IFN-γ and CD56, after excluding the PKH67+ population.

### 2.7 Stable isotope labelling by amino acids in cell culture (SILAC) experiment

SILAC RPMI 1640 media (Gibco, Life Technology) was supplemented with 10% heat-inactivated dialysed Fetal Bovine Serum and 2 mM L-glutamine, and isotopically labelled by adding either 0.3 mM L-Lysine-^13^C_6_ and 0.3 mM L-Arginine-^13^C_6_ (medium isotope), or 0.3 mM L-Lysine-^13^C_6_,^15^N_2_ and 0.3 mM L-Arginine-^13^C_6_,^15^N_4_ (heavy isotope), all kindly provided by Dr Amy McCarthy, Cancer Research UK, Manchester, United Kingdom. Cells were grown in either medium or heavy RPMI media for 5 passages and a labelling efficiency test was performed to verify that cell labelling had been achieved. Medium-labelled Raji cells (Raji-M) and heavy-labelled Raji cells (Raji-H) were cultured for 24 h on plastic and on a layer of M2-10B4 stromal cells, respectively. Cells were separated by FACS sorting, lysed as previously described and protein content was measured by BCA assay. 10 μg Raji-M and 10 μg Raji-H were mixed 1 to 1, resolved by SDS-PAGE and analysed by mass spectrometry (Supplementary Material).

### 2.8 Microscopy and high-content screening

For re-organisation of the actin cytoskeleton in the presence or absence of stromal cells, cells were imaged in 24-well plates under a Zeiss lowlight microscope (×10 air objectives). For time-lapse experiments, cells were kept in an environmental chamber at 37°C, 5% CO_2_ and imaged every 30 min for 24 h with a Zeiss lowlight microscope (×20 air objective). Videos were developed using the MetaMorph Microscopy Automation and Image Analysis Software (Molecular Devices). For characterisation of the actin cytoskeleton and of the CD20 molecule in the presence or absence of stromal cells, mCherry-M2-10B4 cells were plated in CellCarrier-96 Black, Optically Clear Bottom plates (Perkin Elmer). Raji-GFP-actin cells or Raji-YFP-CD20 cells were added to the culture for 24 h. Cells were then centrifuged, labelled with Hoechst 33,342 (0.1 μg/mL) by incubating the cells for 1 h at 37^o^C and imaged under an Opera Phenix High-Content Screening System (Perkin Elmer, X40 water immersion objective). Images were taken in confocal mode and analysed by using the software Harmony High-Content Imaging and Analysis Software (Perkin Elmer) and Columbus Image Data Storage and Analysis System (Perkin Elmer).

### 2.9 Data analysis and statistics

Flow cytometric data were analysed by using FlowJo (Tree Star, Inc.). Graphs and statistical analyses were performed by using two-way Anova in GraphPad Prism. Differences were deemed significant when *p* < 0.05. Data are the average ±SEM of three independent experiments each performed in duplicates or triplicates, unless differently stated in figure legends. In IPA *p*-values are calculated using a right-tailed Fisher’s Exact Test. In DAVID *p*-values are calculated using a modified Fisher’s Exact Test, namely, EASE score. Statistical significance was *p* < 0.05 or ES > 1.3.

## 3 Results

### 3.1 The presence of stromal cells protects B-cell lymphoma cells from obinutuzumab-induced cell death

**FIGURE 1:**
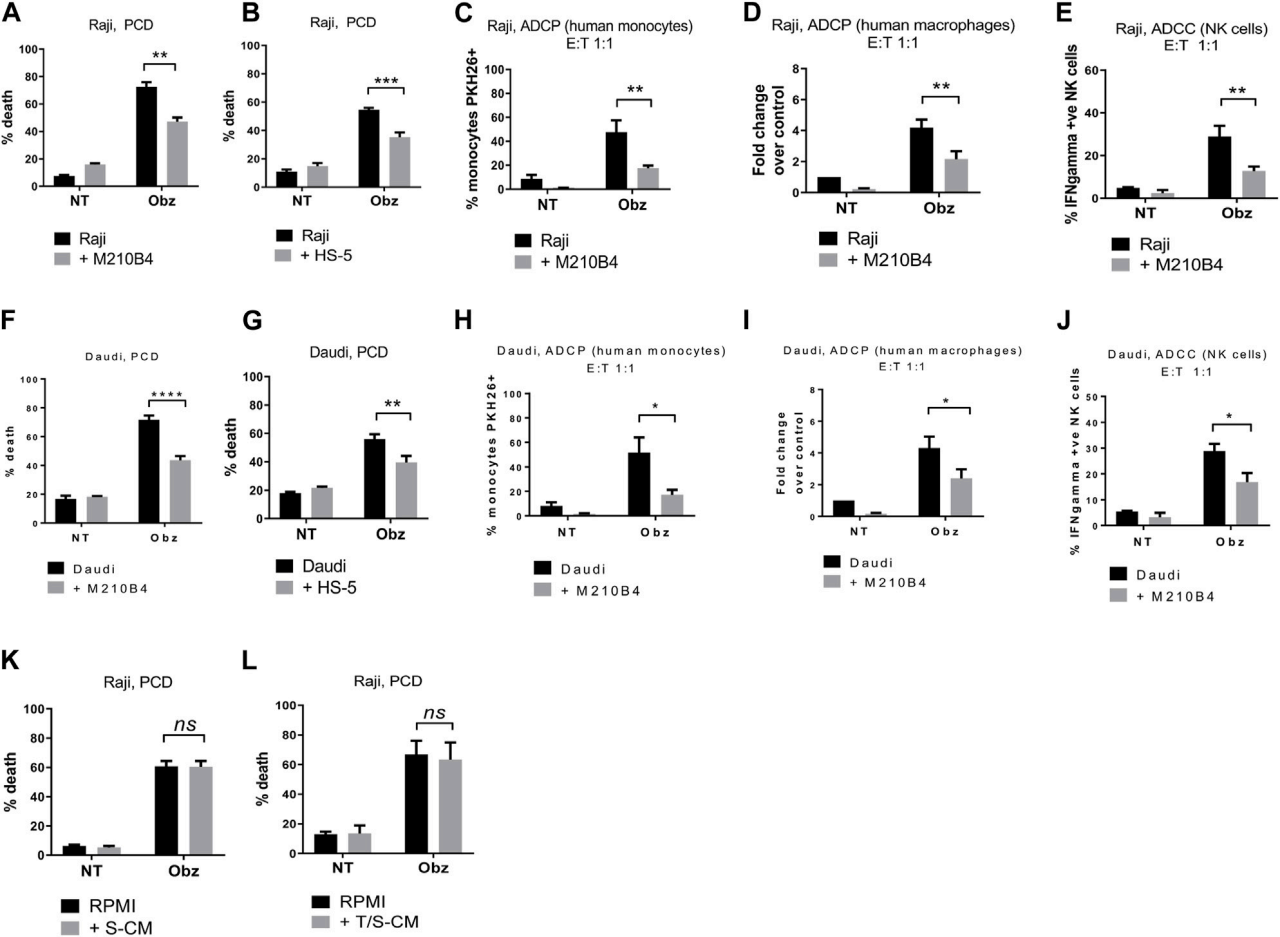
**(A)** Raji cells were cultured either on plastic (black bars) or on a layer of M2-10B4 murine stromal cells (grey bars) for 1 h and either treated with obinutuzumab (Obz) at 10 μg/mL, or left untreated (NT) for 23 h. Survival percentages were calculated by labelling cells with 7-AAD and AnnexinV. **(B)** The same experiment was repeated with the human stromal line HS-5. **(C)** Human monocytes were isolated from PBMCs and added to the co-culture (E:T ratio = 1:1) for 24 h. Percentage phagocytosis were measured as percentage of PKH26-labelled tumour cells which were CD11b^+^. **(D)** Human macrophages were isolated from PBMCs, differentiated by addition of M-CSF and added to the co-culture (E:T ratio = 1:1) for 24 h. Percentages of phagocytosis were measured as percentage of PKH26-labelled tumour cells which were CD11b^+^. **(E)** Human NK cells were isolated from PBMCs and added to the co-culture (E:T ratio = 1:1) for 24 h. Percentages of NK cell activation were measured as percentage of PKH26-labelled tumour cells which were IFNγ^+^. **(F–J)** The experiments were repeated with the B-cell lymphoma cell line Daudi. **(K)** Raji cells were cultured either in RPMI media (black bars) or M2-10B4-conditioned media (S-CM, grey bars) for 1 h and either treated with obinutuzumab (Obz) at 10 μg/mL or left untreated (NT) for 23 h. Survival percentages were calculated by labelling cells with 7-AAD and AnnexinV. **(L)** Raji cells were cultured either in RPMI media (black bars) or in a Raji/M2-10B4-conditioned media (T/S-CM, grey bars) for 1 h and either treated with obinutuzumab (Obz) at 10 μg/mL or left untreated (NT) for 23 h. Survival percentages were calculated by labelling cells with 7-AAD and AnnexinV. Data is mean ± SEM of at least 3 independent experiments.

To determine whether the presence of stroma could influence obinutuzumab-induced killing of B-cell lymphoma tumour cells, Burkitt’s lymphoma Raji cells were cultured either on plastic or on a layer of murine bone marrow M2-10B4 stromal cells. Cells were treated with 10 μg/mL obinutuzumab for 24 h and then harvested and cell death quantified using 7-AAD/AnnexinV staining. Whilst Raji cells were efficiently killed by obinutuzumab when cultured on plastic (72% ± 3% dead cells), the presence of stromal cells significantly reduced the killing to 47% ± 3% (Figure 1A, *p* = 0.0022). A similar decrease in cell death was observed when culturing Raji cells on a layer of human bone marrow stromal cells HS-5 (Figure 1B, *p* = 0.0006). To determine whether the presence of stromal cells could also influence obinutuzumab-induced ADCP, Raji cells were cultured either on plastic or on a layer of M2-10B4 cells for 24 h. Human monocytes were added to the co-culture and wells were treated with obinutuzumab for 2 h. The percentage of monocytes that had engulfed Raji cells cultured on plastic was 48% ± 10, however, this was significantly decreased to 17% ± 2 when Raji cells were cultured on stromal cells (Figure 1C, *p* = 0.0076). Similar results were observed using differentiated human macrophages as effector cells (Figure 1D, *p* = 0.0092). Next, the influence of stroma on obinutuzumab-induced ADCC was determined by measuring the production of IFN-γ by NK cells. NK cells were isolated from PBMCs and added to the co-culture system. After a 4-h treatment with obinutuzumab, the levels of activated (IFN-γ-producing) NK cells was 29% ± 5, but this decreased to 13% ± 2 when cells were cultured in the presence of stromal cells (Figure 1E, *p* = 0.0078). These observations were replicated when studies were performed in a second Burkitt’s lymphoma line, Daudi (Figures 1F–J) and PCD by the type II anti-CD20 antibody tositumomab and the type I antibody rituximab was also inhibited in the presence of stromal cells (Supplementary Figures S1I, S1J). To understand whether such a protective effect from obinutuzumab could also be mediated by the ECM component fibronectin (FN), tumour cells were treated with obinutuzumab either on plastic, or on stroma, or in FN-coated wells. However, the presence of FN could not recapitulate the effect observed with M2-10B4 (Supplementary Figures S1A, S1B). Finally, we sought to determine whether soluble factors played any roles on the protective effect mediated by stromal cells. Raji cells were cultured with conditioned media released by either stromal cells on their own (S-CM) or by stromal cells after a 24 h culture with tumour cells (T/S-CM) treated with obinutuzumab for 24 h and the percentage of death for Raji cells cultured either in normal RPMI media or in S-CM (Figure 1K) and T/S-CM (Figure 1L) was measured by labelling cells with 7-AAD/AnnexinV. Interestingly, no differences were observed between the efficacy of obinutuzumab at inducing PCD when cells were treated in normal RPMI media vs*.* S-CM (*p* = 0.9993) or T/S-CM (*p* = 0.9472), suggesting that the protective effect is not dependent on soluble factors produced by M2-10B4 stromal cells. The same results were obtained with Daudi cells (Supplementary Figures S1C, S1D). In addition, ADCP and ADCC were not affected by culturing tumour cells in S-CM or T/S-CM (Supplementary Figures S1E–H).

### 3.2 Protection from obinutuzumab-induced cell death is dependent on direct contact between stromal and tumour cells

**FIGURE 2:**
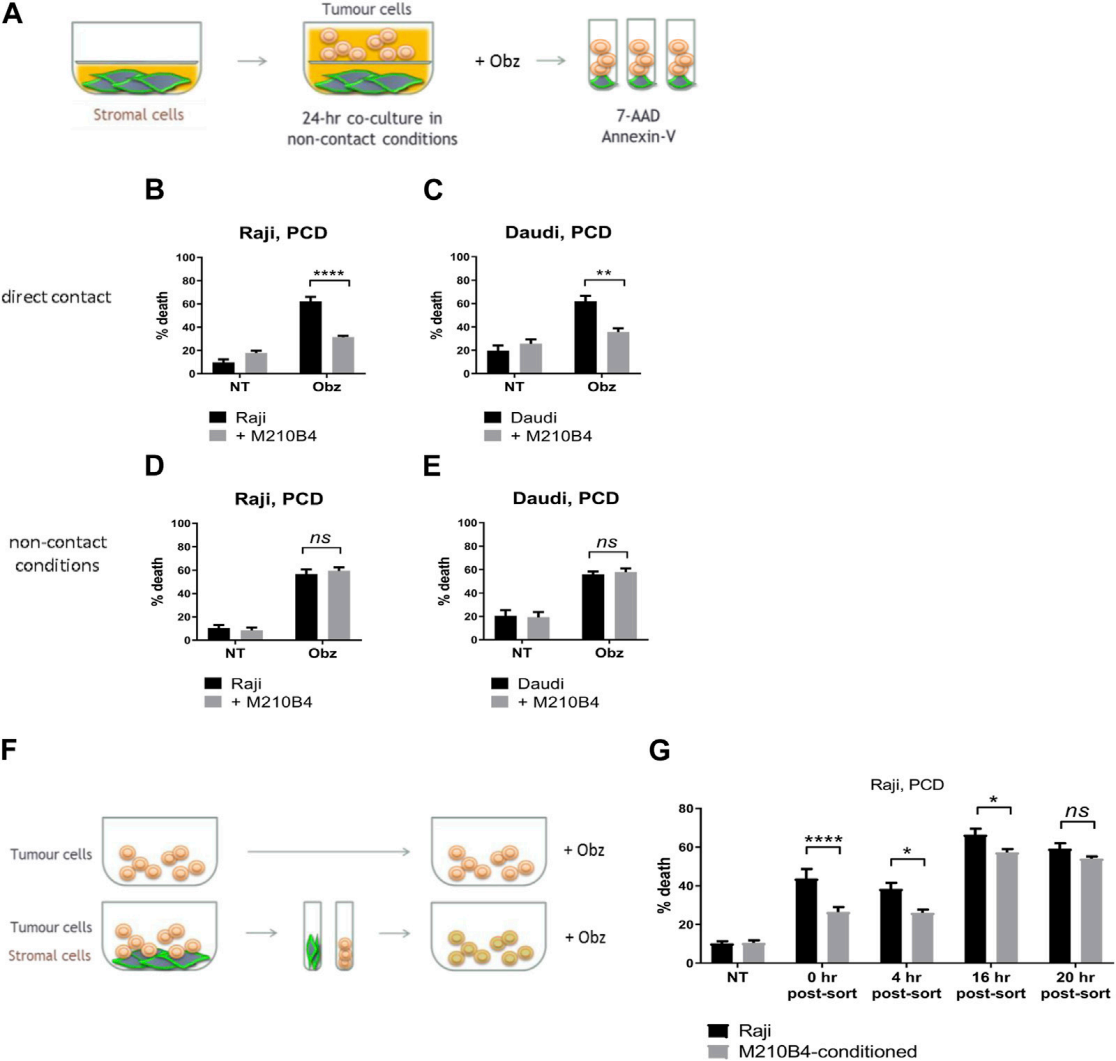
**(A)** Stromal cells were cultured on the bottom compartment of a transwell plate. Tumour cells were added to the upper compartment for 1 h and treated with Obz for 23 h. Survival percentages in direct contact conditions [shown in **(B**) for Raji, **(C)** for Daudi] were calculated by labelling cells with 7-AAD and AnnexinV. Survival percentages for a control assay, were cells were plated in non-contact conditions with the stromal layer, are shown in **(D)** for Raji and **(E)** for Daudi. **(F)** Tumour cells were cultured either on plastic or on a layer of PKH67-labelled M2-10B4 cells for 24 h. Cells were separated through the use of a FACS sorter and PKH67^-^ tumour cells were plated on plastic. Cells were treated with Obz on plastic for further 4 h either immediately after sort (0 h post-sort) or 4, 16 or 20 h post-sort. Survival percentages, shown in **(G)**, were calculated by labelling cells with 7-AAD and AnnexinV. Data is mean ± SEM of two to four independent experiments.

To investigate whether the protective effect from obinutuzumab-induced cell death was dependent on direct contact between stromal and tumour cells, a transwell assay was performed. As shown in Figure 2A, microporous inserts of 0.4 μm pore size were employed to separate the bottom compartment of wells, where M2-10B4 cells were grown, from the upper compartment, where tumour cells were cultured and treated with obinutuzumab for 24 h. Such a pore size was chosen so that media and soluble factors could cross the barrier, but not cells. Whilst Raji cells cultured in normal wells, and therefore in direct contact with M2-10B4 stromal cells, were protected from obinutuzumab-induced PCD (Figure 2B, *p* < 0.0001), Raji cells cultured in non-contact conditions were efficiently killed by obinutuzumab (Figure 2D, *p* = 0.7759), despite the presence of stromal cells in the bottom compartment of transwell plates. The same results were obtained with Daudi cells (Figures 2C, E), further indicating that direct contact between stroma and tumour cells is necessary to achieve protection from obinutuzumab. To determine whether the protection mediated by contact between stromal and tumour cells could also be observed after removal of direct interaction, Raji cells were cultured either on plastic or on PKH67-labelled M2-10B4 stromal cells for 24 h. Cells were then separated by FACS sorting and purified Raji cells were collected, cultured on plastic and treated with obinutuzumab for 4 h at the following times: immediately after sort (0 h post-sort) or 4 h, 16 h, 20 h post-sort (Figure 2F). Intriguingly, a protection from obinutuzumab-induced PCD was still visible in cells that were previously grown on stroma (M2-10B4-conditioned) and treated on plastic immediately after removal of direct contact (Figure 2G, 0 h post-sort, *p* < 0.0001). Furthermore, this protective effect was observed for up to 16 h post-sort but disappeared within 20 h. These data suggest that the protective effects of signalling pathways which are activated by stromal cell contact persist for at least 16 h after removal of direct interaction.

### 3.3 Interaction with stroma influences obinutuzumab-induced homotypic adhesion of tumour cells

**FIGURE 3:**
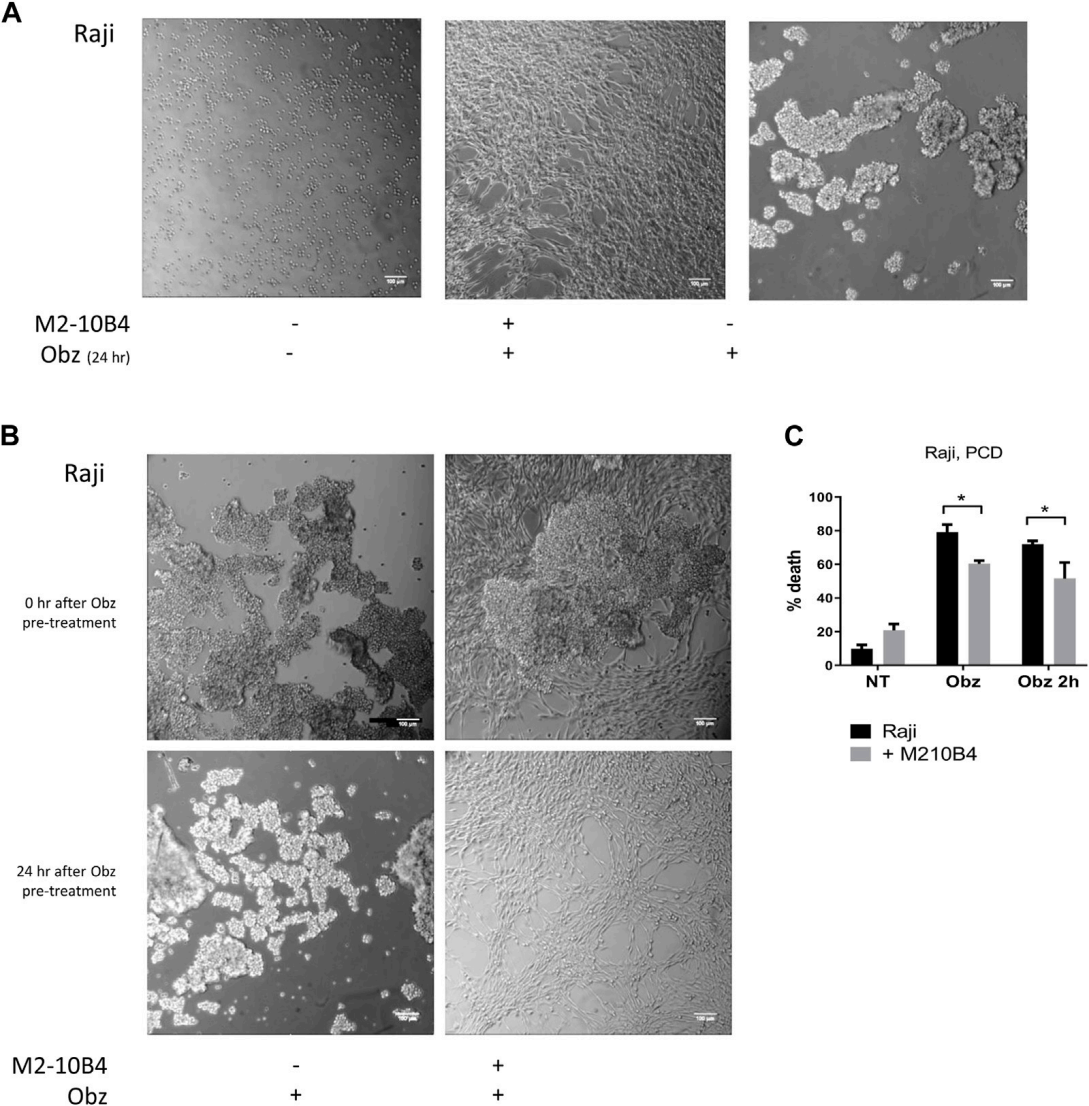
**(A)** Raji cells were cultured either on plastic (left panel) or on a layer of M2-10B4 stromal cells (middle panel) and treated with Obinutuzumab (Obz) at 10 μg/mL. Images were taken on a Zeiss lowlight microscope (10X) 24 h later. Non-treated Raji cells cultured on plastic are shown in the right panel. **(B)** Raji cells were pre-treated with Obz in tubes for 2 h and poured onto either plastic (left panels) or onto a layer of M2-10B4 stromal cells (right panels). Images were taken on a Zeiss lowlight microscope (10X) either straight after the pouring (upper panels) or 24 h later (lower panels). **(C)** Raji cells were cultured either on plastic (black bars) or onto a layer of M2-10B4 stromal cells (grey bars) and either left untreated (NT) or treated with Obz at 10 μg/mL (Obz) or pre-treated with Obz at 10 μg/mL for 2 h (Obz 2h) in a tube and then poured onto the wells. Survival percentages were calculated by labelling cells with 7-AAD and AnnexinV. Data is mean ± SEM of at least 3 independent experiments.

Obinutuzumab-induced PCD involves a process of actin re-organisation and formation of HA between tumour cells (Alduaij et al., 2011; Honeychurch et al., 2012). We sought to understand whether the presence of stromal cells could affect such a process and thus hamper the initiation of PCD. First, tumour cells were cultured either on plastic or on M2-10B4 stromal cells and were either left untreated or treated with obinutuzumab. 24 h later, cells were imaged under a lowlight microscope. As expected, obinutuzumab-treated Raji cells cultured on plastic underwent HA and formed B-cell aggregates (Figure 3A, right panel). However, when cultured on M2-10B4 stromal cells, obinutuzumab-treated Raji cells appeared organised as single cells and attached to the stromal layer (fibroblast-like stellate cells, Figure 3A middle panel) and resembling untreated cells (Figure 3A, left panel). To understand whether HA could also be reversed by stromal cells Raji cells were pre-treated with obinutuzumab for 2 h. The B-cell aggregates were then poured onto a layer of M2-10B4 stromal cells, and images were taken either immediately after addition or 24 h later. After the 2-h pre-treatment with obinutuzumab, Raji cells poured either onto plastic or onto stroma both displayed HA and formation of cell aggregates (Figure 3B, upper panels). Whilst the aggregates were still present in Raji cell cultured on plastic 24 h later (Figure 3B, lower panel, left), aggregated Raji cells cultured in the presence of stromal cells appeared to have disaggregated and returned to normal morphology (Figure 3B, lower panel, right). These data suggest that the contact with stromal cells can reverse HA and B-cell aggregation initiated upon obinutuzumab treatment. This was mirrored by a reduction in the percentage cell death when treatment was started in co-culture conditions (“Obz,” *p* = 0.0464), and when lymphoma cells were pre-treated and then added to the stromal layer (“Obz 2h,” Figure 3C, *p* = 0.0299). A similar reduction in the percentage of death in pre-treated cells when co-cultured with M2-10B4 was also observed with Daudi cells (Supplementary Figure S2A). To further confirm the finding that HA is reversed in the presence of stroma, a time-lapse analysis was performed. Raji cells were pre-treated with obinutuzumab for 2 h and added to a layer of M2-10B4 stromal cells. Pictures of the wells were then taken every 30 min for 24 h. Whilst at time 0 (immediately after addition) aggregated cells are clearly visible (Supplementary Figure S2B, top left), cells re-organise over time, and B lymphoma cells detach from each other by 24 h (Supplementary Figure S2B, bottom right), suggesting that actin reorganisation is able to reverse HA.

### 3.4 Stromal interaction reduces actin cytoskeleton reorganisation and CD20 relocalisation in response to obinutuzumab

**FIGURE 4:**
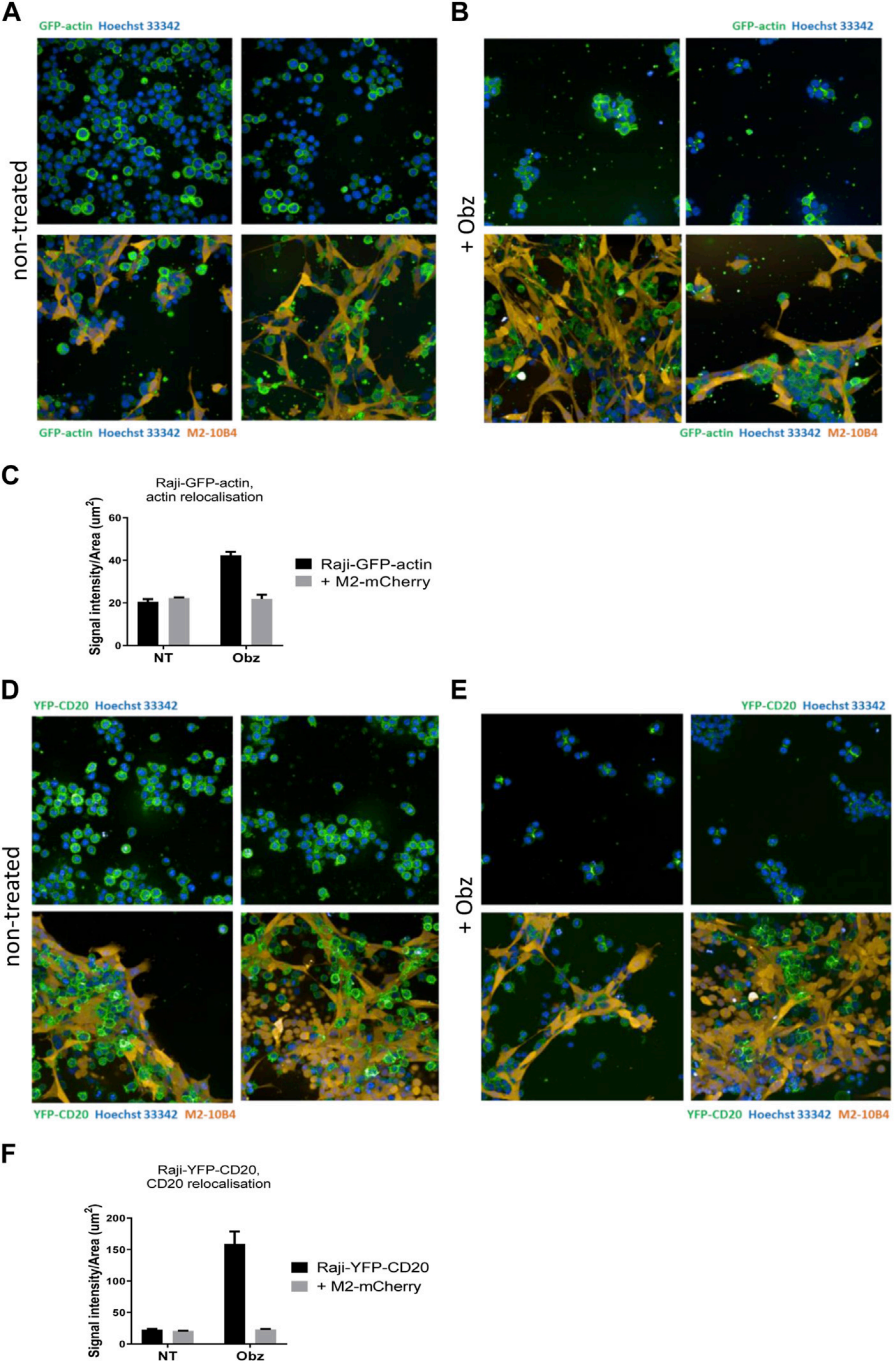
**(A,B)** Raji-GFP-actin cells (green) were cultured either on plastic (upper panels) or on a layer of mCherry-M2-10B4 stromal cells (orange, lower panels) and either left untreated **(A)** or treated with Obz at 10 μg/mL **(B)** for 24 h. Nuclei (blue) were labelled with Hoechst 33,342 (0.1 μg/mL) and plates were scanned on a PerkinElmer Opera Phenix (20X) 24 h later. **(C)** The signal intensity to area ratio for Raji-GFP-actin cells was calculated using Columbus software. **(D–E)** Raji-YFP-CD20 cells (green) were cultured either on plastic (upper panels) or on a layer of mCherry-M2-10B4 stromal cells (orange, lower panels) and either left untreated **(D)** or treated with Obz at 10 μg/mL **(E)** for 24 h. Nuclei (blue) were labelled with Hoechst 33,342 (0.1 μg/mL) and plates were scanned on a PerkinElmer Opera Phenix (20X) 24 h later. **(F)** The signal intensity to area ratio for Raji-YFP-CD20 cells was calculated using Columbus software. **p < 001 unpaired *t*-test. Data is mean ± SEM of at least 3 independent experiments.

To better visualise the actin re-organisation of Raji cells in the presence of stroma, Raji-GFP-actin cells were used. An M2-10B4 stromal line expressing mCherry, (M2-mCherry), was generated and used instead of the parental M2-10B4 line. Raji-GFP-actin cells were cultured either on plastic or on a layer of M2-mCherry for 24 h. Then, nuclei were labelled with Hoechst 33,342 and wells were scanned by high-content screening. Whilst the actin cytoskeleton was evenly distributed around the nuclei in non-treated Raji-GFP-actin cells (Figure 4A, upper panels), HA and movement of the actin signal towards junction points between cells was observed in obinutuzumab-treated cells (Figure 4B, upper panels), as previously shown in Honeychurch et al. (2012). In the presence of M2-mCherry cells, however, Raji-GFP-actin cells appeared attached to the stromal layer and the shape of their actin cytoskeleton did not change following obinutuzumab treatment (Figure 4B, lower panels). This behaviour is reflected by the signal intensity to signal area ratio, which is higher for cells having a similar or higher intensity contained in a smaller area. On plastic (black bars) the ratio increased significantly from 20.5 (untreated cells) to 42.2 (obinutuzumab-treated cells). However, in the presence of stroma (grey bars), there was no statistically significant difference between untreated and obinutuzumab-treated cells (Figure 4C). We sought to determine whether treatment with obinutuzumab could also lead to re-organisation and movement of the CD20 molecule towards the cell-cell junction points. Raji-YFP-CD20 cells were treated as described for Raji-GFP-actin cells and nuclei were labelled with Hoechst 33,342 before scanning wells by high-content screening. In non-treated cells, the CD20 molecules are all evenly disposed on the membrane of tumour cells (Figure 4D, upper panels). When cultured on stroma, Raji-YFP-CD20 cells appear to attach to M2-10B4 stromal cells (Figure 4D, lower panels). Similarly to what was observed with the actin cytoskeleton, treatment with obinutuzumab led to a re-localisation of the CD20 molecules towards the cell-cell junction points, with Raji-YFP-CD20 cells now undergoing HA and aggregating in clumps (Figure 4E, upper panels). When the treatment was performed in the presence of stroma, however, this CD20 re-localisation is reduced, and Raji-YFP-CD20 cells appear attached to the stromal layer instead (Figure 4E, lower panels). Again, this is reflected by the signal intensity to signal area ratio, with an increased ratio only observed in obinutuzumab-treated cells cultured on plastic, but not on stroma (Figure 4F).

### 3.5 Direct contact with stroma leads to downregulation of CD20 on the surface of tumour cells

**FIGURE 5:**
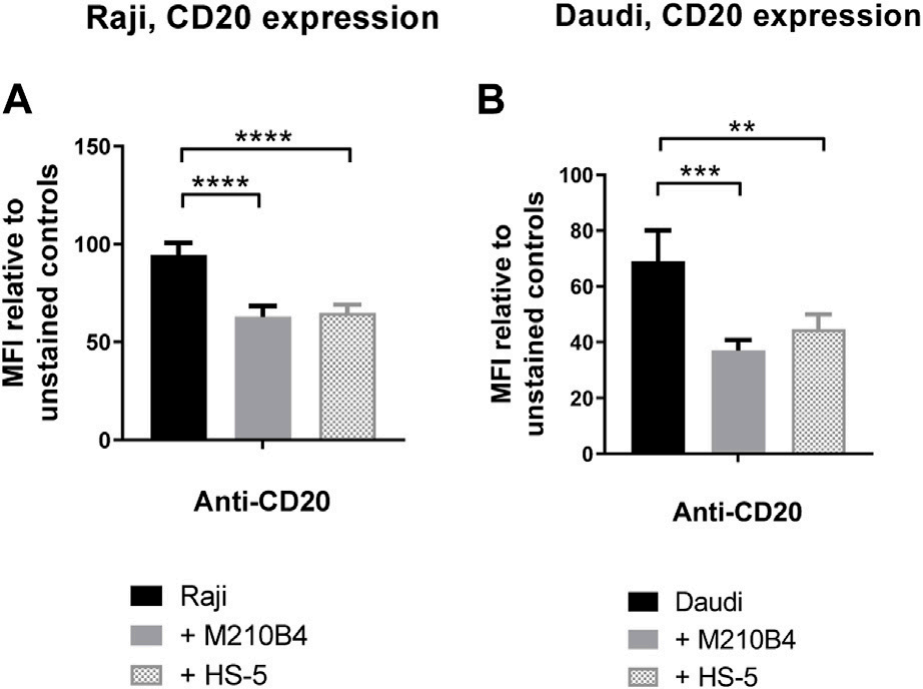
**(A,B)** Co-culture of tumour cells with stromal cells significantly decreases surface levels of the CD20 molecule. Raji **(A)** and Daudi **(B)** cells were cultured for 24 h on either plastic (black bars) or M2-10B4 (gray bars) or HS-5 (checked bars). After 24 h, cells were collected and either left unlabelled, or incubated with isotype control (mouse IgG2b-APC), or anti-CD20-APC antibody (0.24 μg/mL). Percentage of positivity to the antibodies was measured as % of PKH67- cells which were APC+. The MFI (geometric mean fluorescence intensity) measured for isotype controls was then subtracted by the MFI of stained samples. Data is mean ± SEM of three independent experiments performed in duplicate.

Given the finding that interaction with stromal cells was able to inhibit the obinutuzumab-mediated re-modelling of CD20 on the surface of Raji-YFP-CD20 cells, and that a reduction in surface levels of CD20 was previously observed upon culture of Raji cells with HS-5 stromal cells (Marquez et al., 2015), we sought to determine whether the contact with stroma led to a decrease of CD20 level in our model. To address this question, Raji and Daudi cells were cultured for 24 h in the presence of either M2-10B4 or HS-5 stromal cell lines, and CD20 expression was analysed by flow cytometry at the end of the culture. Surface levels of CD20 were significantly decreased after culture with stromal cells, compared to culture on plastic (Raji: *p* < 0.0001 for both stromal lines; Daudi: *p* = 0.0005 for M2-10B4, *p* = 0.0083 for HS-5) (Figures 5A, B; Supplementary Figure S3), suggesting that the reduced ability of obinutuzumab to induce cell death might be due to a downregulation of the target CD20 molecule.

### 3.6 Treatment with ibrutinib further decreases CD20 expression level and obinutuzumab-induced PCD

**FIGURE 6:**
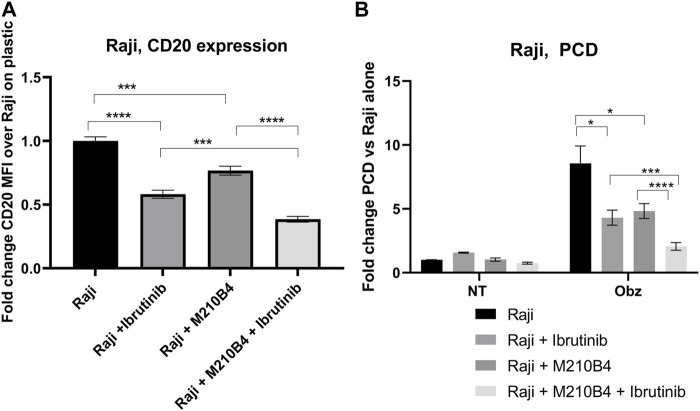
**(A,B)** Effect of pre-treatment with ibrutinib on CD20 expression levels and obinutuzumab-induced PCD. Raji cells were either left untreated (circles), or pre-treated with ibrutinib for 1 h (squares), and then cultured in the presence of M2-mCherry (triangles) and 10 μg/mL obinutuzumab [all four conditions **(B)**] for further 24 h. After 24 h, cells were collected and either left unlabelled, or incubated with isotype control (mouse IgG2b-APC), or anti-CD20-APC antibody (0.24 μg/mL) **(A)**, or labelled with 7-AAD and AnnexinV **(B)** Percentage of positivity to the antibodies was measured as % of mCherry-cells which were APC+. The MFI (geometric mean fluorescence intensity) measured for isotype controls was then subtracted by the MFI of stained samples. Survival percentages were calculated by labelling cells with 7-AAD and AnnexinV. The percentage of cells AnnexinV^+ve^/7-AAD^+ve^ were calculated and are expressed as a fold change relative to untreated Raji cells alone. Data is the mean + SEM of three independent experiments performed in triplicate.

Ibrutinib, a Bruton’s tyrosine kinase (BTK) inhibitor, has been recently introduced for treatment of B cell malignancies, showing success in patients with CLL (Moreno et al., 2019). Despite the improved therapeutic outcomes that were observed which led to ibrutinib being granted FDA approval in combination with obinutuzumab in 2019, several studies have shown contrasting results with regards to the effect of ibrutinib on the efficacy of anti-CD20 mAb (Bojarczuk et al., 2014; Da Roit et al., 2015). Interestingly, Bojarczuk and colleagues suggested that the diminished efficacy of anti-CD20 mAb in killing tumour B cells could be due to a decrease in CD20 expression level on the surface of such cells upon treatment with ibrutinib (Bojarczuk et al., 2014). Against this background, we decided to investigate the effect of a pre-treatment with ibrutinib followed by treatment with obinutuzumab in our system, in order to understand whether a reduction in CD20 expression correlated with a lower efficacy of obinutuzumab at inducing PCD. Raji cells were pre-treated in tubes with 10 μM ibrutinib for 1 h before being transferred onto M2-10B4-mCherry stromal cells or onto plastic. Obinutuzumab was then added at 10 μg/mL for 24 h. After 24 h, both PCD and CD20 expression were measured by flow cytometry. As previously published (Bojarczuk et al., 2014) pre-treatment with ibrutinib led to a significant downregulation of CD20 expression compared to untreated Raji cells on plastic (Figure 6A, *p* < 0.0001, Supplementary Figure S3) and this correlated with a significant reduction in obinutuzumab mediated PCD (Figure 6B, *p* = 0.011). When Raji cells were cultured on M2-10B4, PCD was also efficiently abrogated (Figure 6B, *p* = 0.022) despite a reduction in CD20 levels which was less pronounced than with ibrutinib treatment on plastic (Figure 6A, *p* = 0.0002). Ibrutinib treatment in the presence of M2-10B4 stromal cells led to a greater reduction in CD20 expression than either treatment alone (*p* < 0.000) and further reduced the efficacy of obinutuzumab at inducing PCD compared to treatment with obinutuzumab alone in the presence of M2-10B4 (*p* = 0.0006). These results suggest that contact with stromal cells leads to reduced surface expression of CD20 and decreased obinutuzumab mediated PCD, which could potentially be via a similar mechanism to the ibrutinib mediated reduction in CD20 expression and obinutuzumab mediated PCD. Furthermore, combining ibrutinib treatment of Raji cells with contact with M2-10B4 stromal cells leads to a further reduction in CD20 expression (*p* = 0.0002) and significantly reduced PCD (*p* = 0.003), suggesting that combination treatment with obinutuzumab and ibrutinib would be detrimental, especially in stromal rich tumour areas.

### 3.7 The anti-tumour efficacy of obinutuzumab is lower when treatment is delayed until tumour is established in bone marrow niches

In order to see whether stromal cells might impair obinutuzumab efficacy *in vivo,* a murine model was established where mice received a systemic injection of hCD20^+ve^ lymphoma cells, which led to the development of tumour metastases to spleen and bone marrow and development of hind leg paralysis (Sarzotti et al., 1987; Meunier et al., 2003). Mice were treated with obinutuzumab either 1 day or 7 days after tumour injection (Supplementary Figure S4). Whilst treatment at day 1 led to around 85% long-term survivors, delaying treatment to day 7 (when tumour can be detected by bioluminescent imaging within the mouse femurs, Supplementary Figure S4D) led to only 4 of 14 mice as long-term survivors (28%) suggesting that the close proximity of bone marrow stromal cells to tumour cells might impair obinutuzumab efficacy.

### 3.8 Proteomic analysis of tumour cells in the presence and absence of stroma reveals clusters of pathways which are altered upon co-culture with stromal cells

**FIGURE 7:**
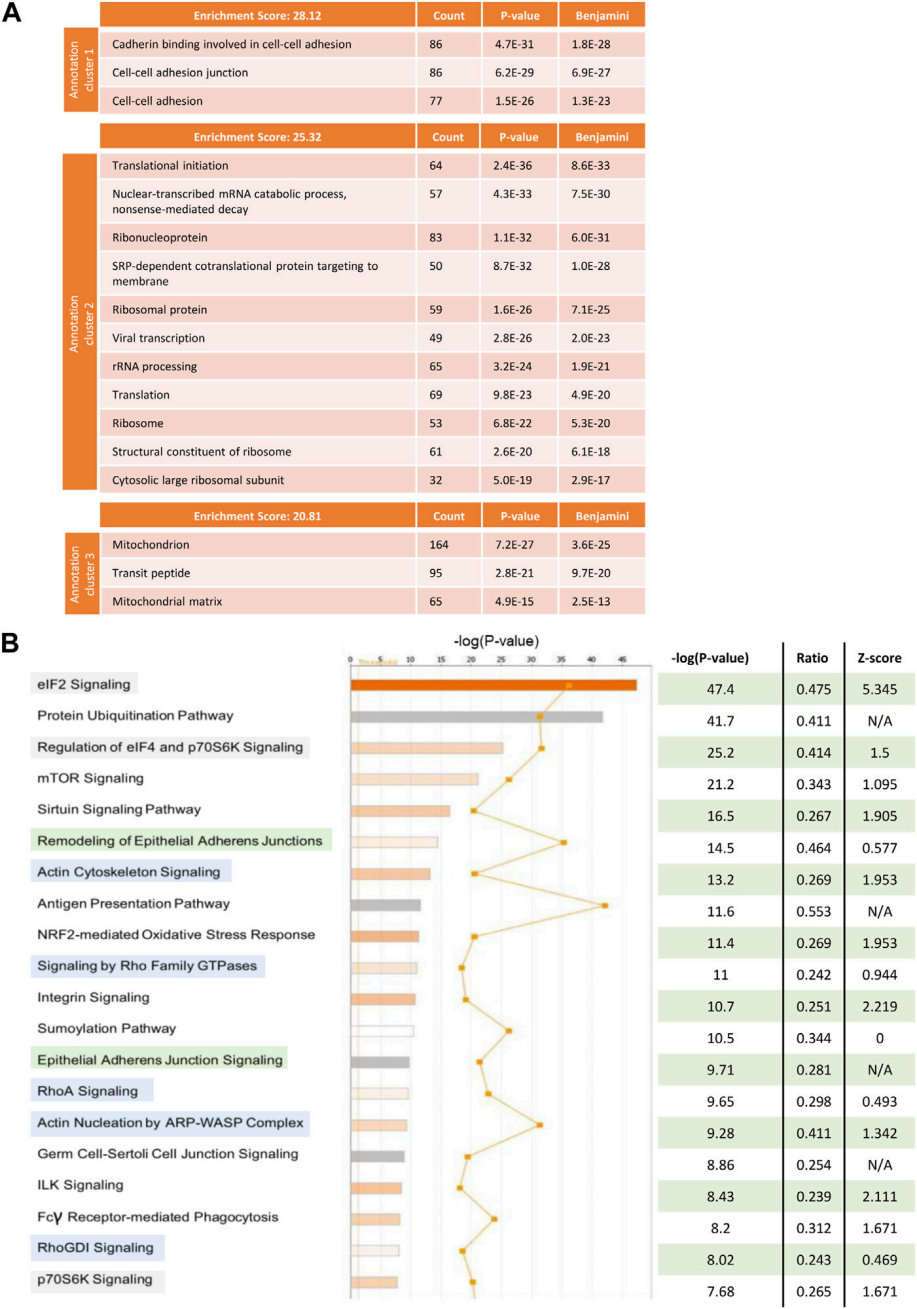
Raji cells were grown in RPMI media containing either heavy (Raji-H) or medium (Raji-M) isotopes of Lysine and Arginine. Cells were cultured either on plastic (Raji-M) or on a layer of PKH67-labelled M2-10B4 stromal cells (Raji-H) for 24 h. Tumour cells were separated from stromal cells by FACS sorting and the resulting populations of Raji-M and Raji-H were lysed. Protein content was calculated and lysates from Raji-M and Raji-H were mixed in a 1 to 1 ratio before being resolved in a SDS-PAGE and analysed by mass spectrometry. **(A)** Protein IDs which had *p*-value <0.05 and had expression fold change < -2 or >2 were uploaded in DAVID. The cluster annotation tool was used to identify the mostly enriched clusters of pathways which were differentially expressed in Raji-H. **(B)** Protein IDs which had *p*-value <0.05 and expression fold change < -2 or >2 were uploaded in IPA. Pathways which were altered in Raji-H compared to Raji-M are shown. For each of them, their significance, ratio and Z-score are shown. Data is from one experiment.

To understand which pathways in tumour cells were being altered by the interaction with stromal cells that could explain the observed effect on obinutuzumab efficacy, a SILAC experiment was performed. Raji cells were grown in a media supplemented with either heavy or medium isotopes of Lys and Arg, as described in Materials and Methods. The resulting isotopically-labelled cell lines Raji-M and Raji-H were cultured on plastic or on a layer of PKH67-labelled M2-10B4, respectively (Supplementary Figure S5). 24 h later, tumour cells were separated from M2-10B4 stromal cells by FACS sorting. Raji-M and Raji-H were lysed and a 1 to 1 mix of the two conditions was resolved on SDS-PAGE. The proteins thus obtained were then run on a mass spectrometer and adjusted *p*-values and expression values were retrieved by running the total protein IDs on PEAKS Studio. All the hits that met the cut-offs “adjusted *p*-value < 0.05” and “expression fold change < −2, >2” were analysed using both DAVID and IPA. In DAVID (Figure 7A), the mostly enriched functional annotation cluster included pathways involved in cadherin-mediated adherens junction signalling (ES = 28.12), translational initiation (ES = 25.32) and mitochondrion (ES = 20.81). IPA also displayed as mostly enriched three pathways involved in translational initiation (Figure 7B, highlighted in green) and two cadherin-mediated adhesion-related pathways (orange). Interestingly, pathways related to the actin cytoskeleton and remodelling were also present (blue). The predicted activation status of each pathway (Z-score) revealed that the majority of the 20 most enriched signalling pathways were also predicted to be upregulated in Raji cultured on stroma compared to Raji cultured on plastic (Figure 7B).

## 4 Discussion

In haematological malignancies, the TME plays an important role in supporting the growth and survival of tumour cells. In CD20^+^ B-cell malignancies, anti-CD20 mAbs have revolutionised therapeutic strategies, however it is currently unclear whether interactions between the TME and anti-CD20 mAb affects treatment outcomes.

Obinutuzumab is a novel glycoengineered type II antibody that only in recent years has been granted FDA approval. Despite the demonstrated superiority of this antibody compared to rituximab in different B-cell malignancies (Goede et al., 2014; Hiddemann et al., 2018), our *in vitro* experiments reveal that the presence of the stromal microenvironment can still significantly reduce killing of tumour cells, affecting the execution of both direct cell death and immune effector-mediated mechanisms of action.

The stromal microenvironment-mediated protection was dependent on direct contact rather than on soluble factors. Although soluble factors have been recognised to play a role in conferring resistance to chemotherapy-induced apoptosis (Kay et al., 2007; Lwin et al., 2007) and the CXCR-4/CXCL-12 signalling axis has been proven instrumental in mediating protection from rituximab (Hu et al., 2012; Beider et al., 2013), other studies have highlighted the importance of direct contact and the relative lack of a role for stroma-conditioned media (Panayiotidis et al., 1996; Edelmann et al., 2008). The discrepancy in these observations could be due to intrinsic differences in the lines and/or samples from different B-cell malignancies tested in those studies. Thus, stroma-mediated drug resistance in different tumour subtypes could be regulated by distinct mechanisms. In addition to this, most published articles protection from either chemotherapeutic drugs or from the type-I antibody rituximab was studied. Obinutuzumab is known to work through distinct mechanisms of action compared to both chemotherapeutic agents and type-I antibodies, suggesting that different mechanisms of protection could be employed by tumour cells against this specific antibody.

As shown by Alduaij et al. (2011), obinutuzumab induces PCD via a non-apoptotic mechanism involving HA between B cells and the reorganisation of the actin cytoskeleton towards the cell-cell junction points. However, in the presence of stromal cells these initial steps leading to PCD were not observed. In fact, stromal cells seem able to interfere with the initiation of PCD by reversing antibody-induced HA of B lymphoma cells. It is not clear how this phenomenon occurs. One hypothesis could be that the interaction between stroma and tumour cells is mediated by the same (set of) receptors involved in obinutuzumab-induced HA between B cell aggregates. Thus, stromal cells could compete for the same adhesion molecules and engage them with a higher affinity, leading to the disruption of B cell aggregates and inhibition of HA. Further investigations into these mechanisms could also help us increase our knowledge of which molecules are involved in the initial steps of obinutuzumab-induced PCD.

Antibody-induced actin cytoskeleton remodelling was also halted by the presence of stromal cells. Tumour cells on plastic adhere to each other forming visible aggregates upon treatment with obinutuzumab, and the actin cytoskeleton relocates towards the cell-cell junction points. However, our high-content screening images reveal that, when cultured on stroma, tumour cells tend to adhere to the stromal layer, and a clear polarisation of the actin signal is absent. Interestingly, a similar pattern of re-localisation–and lack of, when in contact with stroma–was observed for the CD20 molecule. This stroma-dependent alteration in re-localisation of CD20 has not previously been reported, and might suggest that the CD20 molecule could also be involved in stroma-mediated protection from obinutuzumab-induced PCD. Indeed, culture on stromal cells leads to CD20 downregulation and one could hypothesise that reduced surface CD20 might reduce susceptibility to type II anti-CD20 antibodies, as has previously been shown for type I antibodies following CD20 modulation and for ibrutinib treated tumour cells (Beers et al., 2010; Bojarczuk et al., 2014; Tipton et al., 2015; Tomita, 2016). Indeed, killing of CD19^+^ cells in the blood of CLL patients by obinutuzumab was found to directly correlate with surface levels of CD20 (Patz et al., 2011) and downregulation of CD20 through depletion of the transcriptional coactivator CREB binding protein led to reductions in obinutuzumab mediated killing (Scialdone et al., 2019).

The BTK inhibitor ibrutinib was approved by the FDA in 2019 in combination with obinutuzumab in patients with CLL/SLL (Moreno et al., 2019), representing the first instance that anti-CD20 mAbs were employed in a combination treatment that does not include any standard chemotherapeutic agents. The ability of ibrutinib to block adhesion of CLL cells to the TME appeared to be one mechanism that could explain the success of this treatment approach (Herman et al., 2015). Crucially, several studies have since highlighted a detrimental effect of ibrutinib on CD20 expression levels in B-NHL cells, with a subsequent reduction in the efficacy of anti-CD20 mAbs when used in combination therapies (Bojarczuk et al., 2014; Da Roit et al., 2015; Pavlasova et al., 2016). Others have observed a negative effect of ibrutinib on anti-CD20 mAb-mediated ADCC, with decreased ability of NK cells to degranulate and lyse tumour cells (Kohrt et al., 2014). Our data confirmed that ibrutinib mediates downregulation of CD20 expression on the surface of Raji cells with a concomitant decrease in obinutuzumab mediated PCD. Similarly, contact with stromal cells downregulates CD20 expression, and leads to a significant reduction in PCD, suggesting that the level of CD20 on the cell surface might play an important role in the efficacy of anti-CD20 mAbs. Pre-treatment of Raji cells with ibrutinib followed by culture with stromal cells further reduced surface CD20 and obinutuzumab mediated PCD. However, whilst the combination of ibrutinib and obinutuzumab has been shown to be effective in the clinic (Moreno et al., 2019) and *in vivo* models show no loss of obinutuzumab efficacy when ibrutinib is given as a co-treatment (Duong et al., 2015), consideration should be given to whether anti-CD20mAbs and ibrutinib should be given concurrently in the clinic or whether careful scheduling might improve outcomes further.

Proteomic analysis suggested that the presence of stroma could lead to the differential expression of several clusters of pathways. Among these, the cadherin-mediated adherens junction signalling seemed to be the most relevant, as our previous results strongly suggested that interactions between stromal and tumour cells are mediated by direct contact. The expression level of cadherin molecules has previously been reported to be altered in B-cell malignancies (Sharma and Lichtenstein, 2009; Takata et al., 2014), and there is some evidence suggesting that cadherins might play a role in mediating interactions with stromal components in blood malignancies: in T-cell lymphoma, for instance, the expression of N-cadherin enabled interactions of malignant cells with fibroblasts (Kawamura-Kodama et al., 1999). Furthermore, N-cadherin was also shown to be responsible for the protective effect upon contact between CD34^+^ CML cells and mesenchymal stromal cells (Zhang et al., 2013). Therefore, we looked at the expression levels of cadherin molecules in tumour cells cultured on plastic or with stroma, revealing that indeed the presence of stroma caused an upregulation of the R-cadherin molecule (data not shown). However, a CRISPR-Cas9 knock-out of R-cadherin molecule in Raji cells failed to abrogate the stroma-mediated protection from obinutuzumab (data not shown), indicating that the R-cadherin signalling pathway (and its upregulation) was not involved with the observed phenotype.

Our results indicate that the interaction of Burkitt’s lymphoma B cells with the stromal microenvironment negatively influences the efficacy of the anti-CD20 mAb obinutuzumab and suggest that blockade of these interactions could restore obinutuzumab efficacy. Whilst bone marrow derived stromal cells were used in this study it would be important to expand these observations to other types of stromal cells found to interact with B-cell tumours including nodal stromal cells. Several signalling pathways that appeared upregulated in tumour cells upon direct contact with stromal cells were identified, and further studies into which of such candidate pathways is responsible for the stroma-mediated protection from obinutuzumab, and possibly for the additional effect achieved by pre-treatment with ibrutinib in the presence of stroma, are warranted. Pharmaceutical targeting of these pathways could constitute a novel therapeutic strategy that could potentially improve treatment outcomes in patients with B cell malignancies. In the clinical setting, impeding the interactions between stromal and tumour cells might lead to the release into the circulation of those malignant cells which had homed into the protective bone marrow environment. This in turn might be translated into a greater ability of the anti-CD20 mAb obinutuzumab, in combination with standard chemotherapeutic agents, to clear the body of malignant B cells.

## Data Availability

The original contributions presented in the study are included in the article/Supplementary Material, further inquiries can be directed to the corresponding authors.
