# Supplementary material for: Stromal cell inhibition of anti-CD20 antibody mediated killing of B-cell malignancies
Source: Front Cell Dev Biol. 2023 Oct 31;11:1270398. doi: 10.3389/fcell.2023.1270398 (PMC10646167; doi:10.3389/fcell.2023.1270398)
Supplement: Supplementary file 1 [file DataSheet1.pdf]

Supplementary Material

Stromal cell inhibition of anti-CD20 antibody mediated killing of B-cell malignancies

Ester Fagnano[1], Swati Pendharkar[1], Madyson Colton[1], Philip N Jones[1], Marta Crespi Sallan[2], Tetyana Klymenko[3], Andrejs Braun[2], Christian Klein[4], Jamie Honeychurch[1], Eleanor J Cheadle[1*†], Timothy M Illidge[1*†]

*** Correspondence:**

Corresponding Author: eleanor.j.cheadle@manchester.ac.uk

tmi@manchester.ac.uk

1 Supplementary Methods

1.1 Isolation of PBMCs from buffy coats of blood

50 ml buffy coats of blood were diluted 1:4 with PBS and 25 ml of the diluted blood was carefully layered on 20 ml Lymphoprep density gradient media. Tubes were centrifuged at 580g at room temperature for 30 minutes without brake. The cloudy layer containing PBMCs was collected and cells were washed with PBS and centrifuged at 800g at room temperature for 10 minutes with brake. The pellet was re-suspended in a freezing mix containing FBS + 10% DMSO and stored at -80°C until further use.

1.2 Antibody clones

Antibody	Conjugate	Clone	Manufacturer
Anti-hu CD11b	APC	ICRF44	eBioscience, Thermo Fisher Scientific
Anti-hu CD56	APC	TULY56	eBioscience, Thermo Fisher Scientific
Anti-hu IFN-γ	PE	4S.B3	eBioscience, Thermo Fisher Scientific

1.3 Mass spectrometry

Mass spectrometry was performed by the Biological Mass Spectrometry facility at the Cancer Research UK Manchester Institute. Proteins were cleaved by trypsin digestion and the resulting peptides were then subject to nano-liquid-chromatography (LC). A quadrupole/Orbitrap/Ion trap

mass spectrometer (MS) was used to calculate the mass to charge ratio for each peptide's fragment. Peaks Protein Identification Software (Bioinformatic Solutions Inc.) was used to identify each protein based on the mass to charge ratio. Expression fold change for each protein was measured by calculating the ratio between the intensity of analytes in Raji-H and the intensity of analytes in Raji-M.

1.4 *In vivo* experiments

The EL4hCD20 cell line has been described previously (Cheadle et al, 2017) and was modified to express luciferase ires eGFP as described (Cheadle et al, 2010). $5x10^5$ EL4hCD20iGFP cells were injected i.v. into C57Bl/6 mice or NSG mice. Mice received 50µg of obinutuzumab m2a (Cheadle et al 2017, provided by C Klein, Roche Innovation Centre, Zurich) i.p on day 1, 5, 7, 11, 14 or day 7, 11, 14,18, 21. NSG mice were imaged 1 day and 7 days after tumour injection using the Bruker Xtreme imaging system following injection of 150mg/kg Luciferin (Caliper Life Sciences) under anaesthesia. All animal experiments were performed under United Kingdom Home Office Licenses held at the CRUK Manchester Institute, University of Manchester (P8C91D1C5). Mice were housed on aspenchips-2 bedding with enrichment material such as sizzlenest nesting material and cardboard tunnels in a 12/12 light/dark cycle and were given filtered water and fed *ad libitum* on Teklad Global 19% protein extruded rodent diet.

1.5 Supplementary Figures

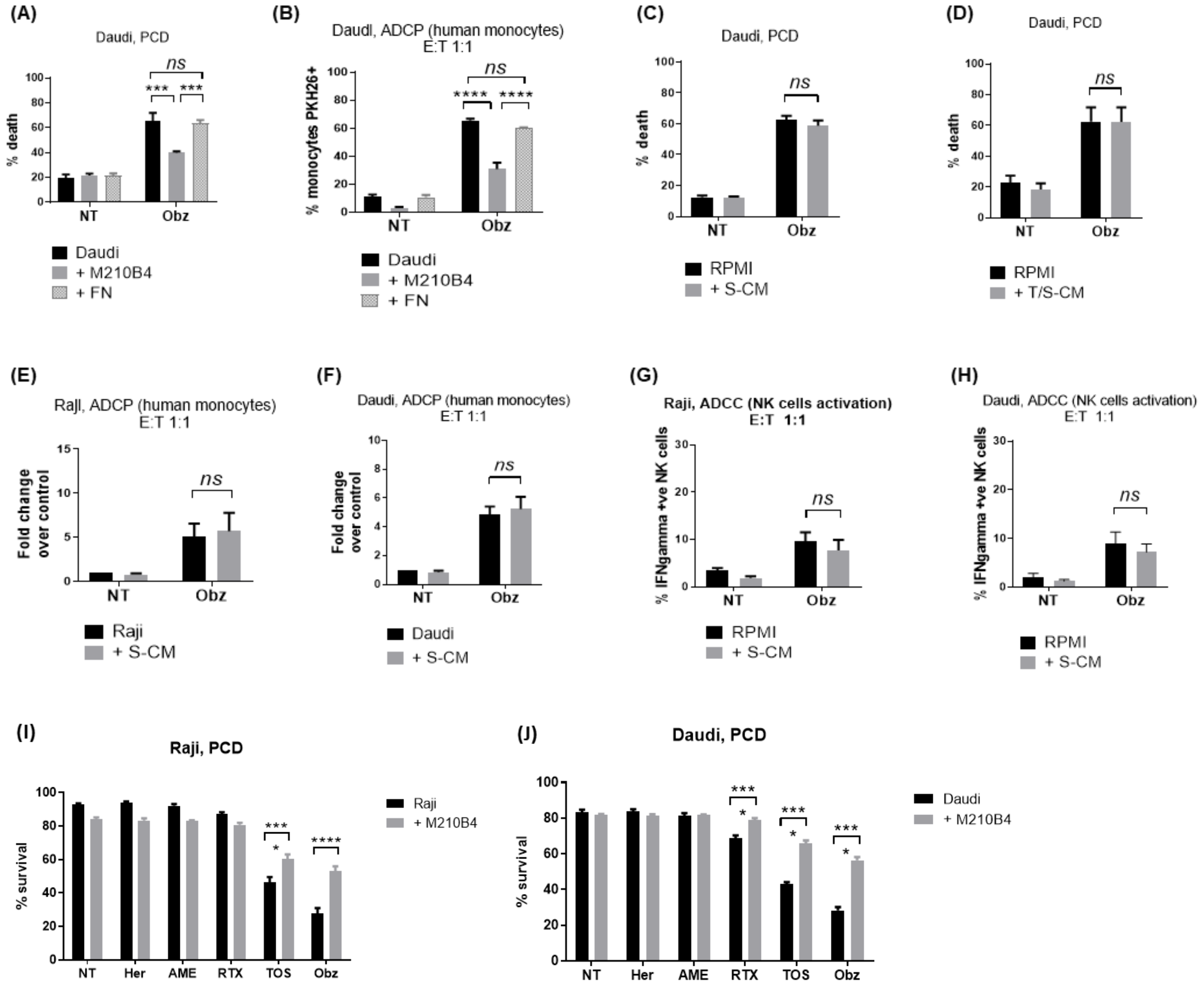

Supplementary Figure 1. (**A).** Daudi cells were cultured either on plastic (black bars) or on a layer of M2-10B4 murine stromal cells (grey bars) or on fibronectin-coated wells (FN, checked bars) for 1 hr and either treated with Obinutuzumab (Obz) at 10 μg/ml, or left untreated (NT) for 23 hr. Survival percentages were calculated by labelling cells with 7-AAD and AnnexinV. (**B).** Human monocytes were isolated from PBMCs and added to the co-culture (E:T ratio = 1:1) for 24 hr. Percentages of phagocytosis were measured as percentage of PKH26-labelled tumour cells which were $CD11b^{+}$. (**C).** Daudi cells were cultured either in RPMI media (black bars) or M2-10B4-conditioned media (S-CM, grey bars) and either treated with Obinutuzumab (Obz) at 10 μg/ml or left untreated (NT) for 24 hr. Survival percentages were calculated by labelling cells with 7-AAD and AnnexinV. (**D).** Daudi cells were cultured either in RPMI media (black bars) or in a Raji/M2-10B4-conditioned media (T/S-CM, grey bars) for 1 hr and either treated with Obinutuzumab (Obz) at 10 μg/ml or left untreated (NT) for 23 hr. Survival percentages were calculated by labelling cells with 7-AAD and AnnexinV. **(E).** Human monocytes were isolated from PBMCs and added to tumour cells cultured either in RPMI or in M2-10B4-conditioned media (E:T ratio = 1:1) for 24 hr. Percentages of phagocytosis were measured as percentage of PKH26-labelled tumour cells which were $CD11b^{+}$. (**F).** Daudi cells were treated as described in E. Percentages of phagocytosis were measured as percentage of PKH26-labelled tumour

cells which were CD11b$^+$. (**G**). Human NK cells were isolated from PBMCs and added to tumour cells cultured either in RPMI or in M2-10B4-conditioned media (E:T ratio = 1:1) for 24 hr. Percentages of NK cell activation were measured as percentage of PKH26-labelled tumour cells which were IFNγ^+. (**H**). Daudi cells were treated as described in G. Percentages of NK cell activation were measured as percentage of PKH26-labelled tumour cells which were IFNγ^+. (**I**) Raji and (**J**) Daudi cells were cultured either on plastic (black bars) or on a layer of M2-10B4 murine stromal cells (grey bars) for 1 hr and either treated with Herceptin, AME-133V, rituximab, tositumomab or obinutuzumab (Obz) at 10 μg/ml, or left untreated (NT) for 23 hr. Survival percentages were calculated by labelling cells with 7-AAD and AnnexinV.

(A)

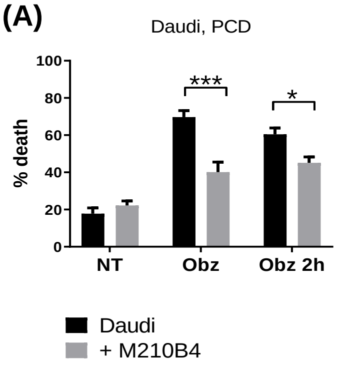

(B)

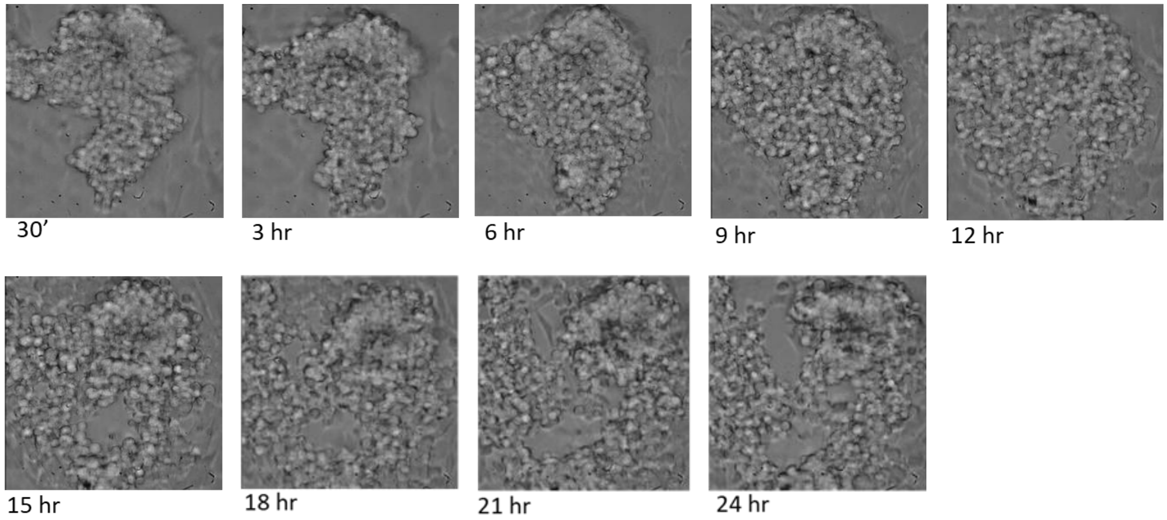

Supplementary Figure 2. (**A).** Daudi cells were cultured either on plastic (black bars) or on a layer of M2-10B4 stromal cells (grey bars) and either left untreated (NT) or treated with Obz at 10 μg/ml (Obz) or pre-treated with Obz at 10 μg/ml for 2 hr (Obz 2h) and then poured onto the wells. Survival percentages were calculated by labelling cells with 7-AAD and AnnexinV. (**B).** Raji cells were pre-treated with Obz for 2 hr and then poured onto a layer of M2-10B4 stromal cells. Pictures were taken every 30min for 24 hr with a Zeiss lowlight microscope (20X) and a film was produced as a result. Still shots of several different time points (as indicated) are shown. Images are representative of 2

independent experiments, each with 5 to 8 fields of view depending on the number of B cell aggregates that were present.

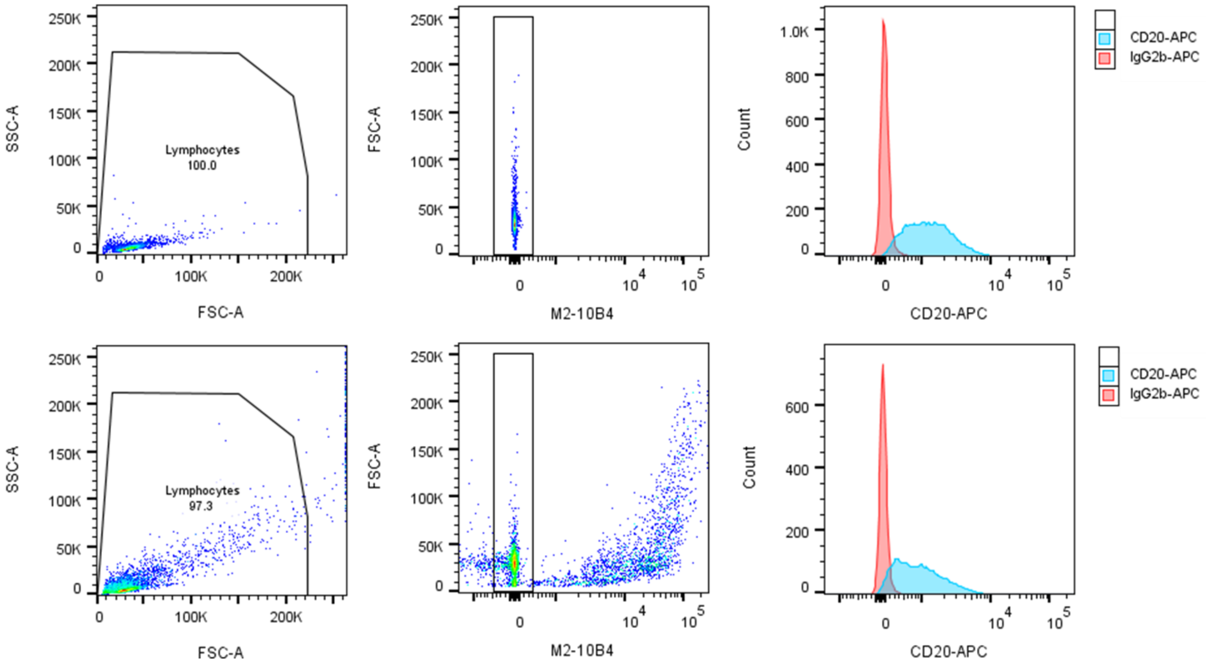

Supplementary Figure 3. Gating strategy for CD20 expression shown in Figures 5 and 6. Cells were gated on FSC/SSC and then M2-10B4 were excluded (PKH26+ or mcherry+). The geometric mean for APC (CD20 or IgG2b isotype control) was then calculated and the MFI for CD20 was calculated as geometric mean CD20 – geometric mean IgG2b for each sample. The fold change of Raji/Daudi cells cultured with stromal cells/Ibrutinib over Raji/Daudi cells cultured on plastic was calculated.

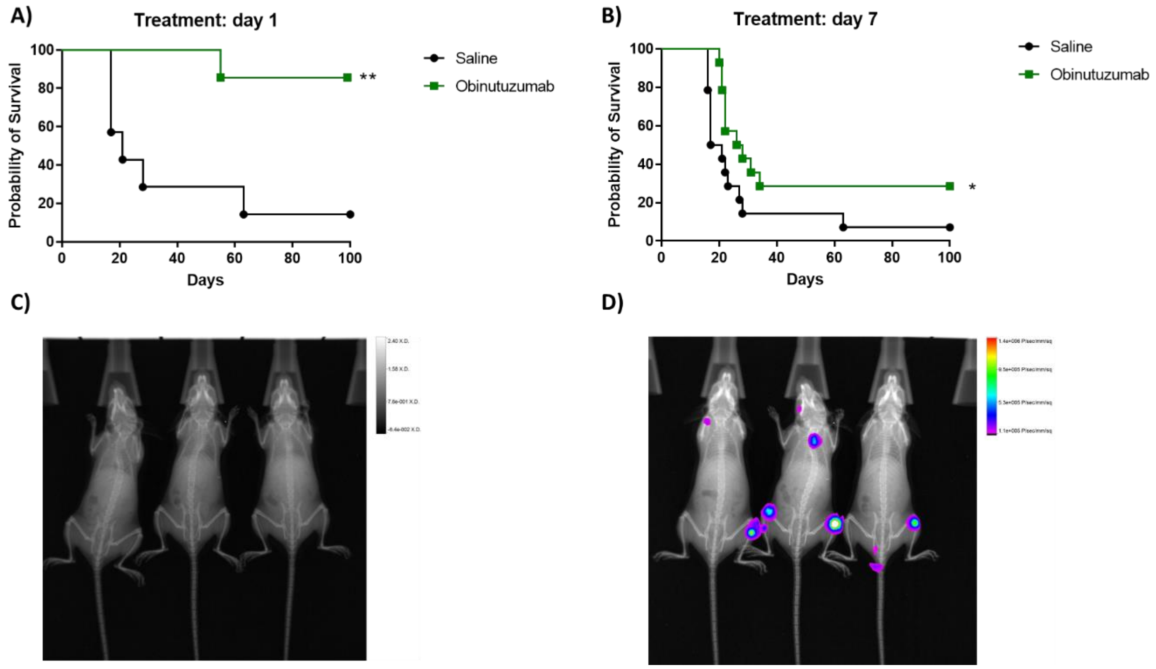

Supplementary Figure 4. A and B) C57bl/6 mice were injected with $5x10^5$ EL4hCD20 i.v. and treated with 50µg obinutuzumab m2a on day 1, 5, 7, 11 and 14 (n=7) (A) or day 7, 11, 14, 18, 21 (n=14) (B). Mice were culled at onset of hind leg paralysis, ill health or palpable tumour masses. C and D) NSG mice were injected with $5x10^5$ EL4hCD20 luciferase iGFP i.v. and imaged on day 1 (C) and day 7 (D) with a Bruker Xtreme imaging system following i.p. administration of luciferin (n=3).

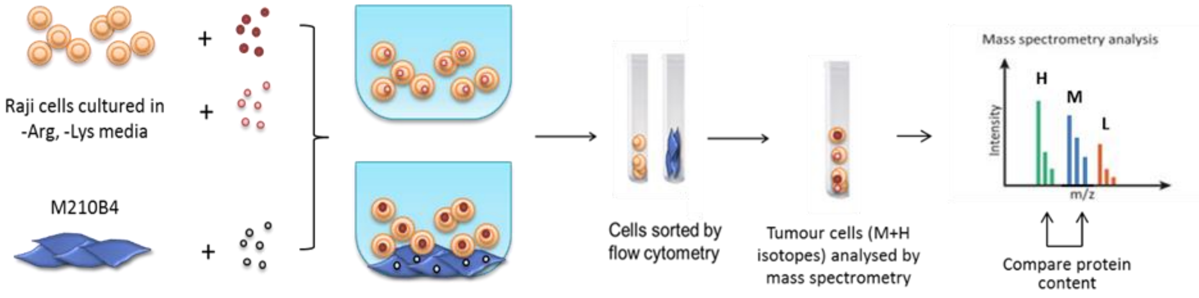

Supplementary Figure 5. Schematic diagram showing the experimental design of the SILAC experiment. Raji cells were grown in RPMI media containing either heavy (Raji-H) or medium (Raji-M) isotopes of Lysine and Arginine. Cells were cultured either on plastic (Raji-M) or on a layer of PKH67-labelled M2-10B4 stromal cells (Raji-H) for 24 hr. Tumour cells were separated from stromal cells by FACS sorting and the resulting populations of Raji-M and Raji-H were lysed. Protein content was calculated and lysates from Raji-M and Raji-H were mixed in a 1 to 1 ratio before being resolved in a SDS-PAGE and analysed by mass spectrometry
